# The Impact of Enzalutamide on the Prostate Cancer Patient Experience: A Summary Review of Health-Related Quality of Life across Pivotal Clinical Trials

**DOI:** 10.3390/cancers13235872

**Published:** 2021-11-23

**Authors:** Bertrand Tombal, Arnulf Stenzl, David Cella, Yohann Loriot, Andrew J. Armstrong, Karim Fizazi, Tomasz Beer, Cora N. Sternberg, Maha Hussain, Cristina Ivanescu, Arijit Ganguli, Krishnan Ramaswamy, Fred Saad

**Affiliations:** 1 Cliniques Universitaires Saint Luc, Avenue Hippocrate, 10, B-1200 Bruxelles, Belgium; bertrand.tombal@uclouvain.be; 2 Department of Urology, Eberhard Karls University of Tübingen, Hoppe-Seyler-Straße 3, 72076 Tübingen, Germany; arnulf.stenzl@med.uni-tuebingen.de; 3 Feinberg School of Medicine, Northwestern University, 633 Clark Street, Evanston, IL 60208, USA; d-cella@northwestern.edu; 4 Institut Gustave Roussy, 114 Rue Edouard Vaillant, Villejuif CEDEX, 94805 Paris, France; yohann.loriot@gustaveroussy.fr (Y.L.); karim.fizazi@gustaveroussy.fr (K.F.); 5 Duke Cancer Institute Center for Prostate & Urologic Cancers, Duke University, 905 La Salle Street, GSRB1 Room 3006, Durham, NC 27710, USA; andrew.armstrong@duke.edu; 6 Oregon Health & Science University Center for Health & Healing, 3485 S. Bond Avenue, Building 2, Portland, OR 97239, USA; beert@ohsu.edu; 7 Division of Hematology and Oncology, Englander Institute for Precision Medicine, Weill Cornell Medicine, Belfer Research Building, 413 East 69th Street, Room 1412, New York, NY 10021, USA; cns9006@med.cornell.edu; 8 Robert H. Lurie Comprehensive Cancer Center, Feinberg School of Medicine, Northwestern University, 303 East Superior Street, Suite 3-107, Chicago, IL 60611, USA; maha.hussain@northwestern.edu; 9 IQVIA, Diana Building, Office 314, Herikerbergweg 181, Zuidoost, 1101 CN Amsterdam, The Netherlands; cristina.ivanescu@quintiles.com; 10 Astellas Pharma Global Development, Inc., 1 Astellas Way, Northbrook, IL 60062, USA; 11 Pfizer Oncology, 235 East 42nd Street, Mailstop 219/06/32, New York, NY 10017, USA; krishnan.ramaswamy@pfizer.com; 12 University of Montréal Hospital Center (CHUM), Montréal Cancer Institute, University of Montréal, Pavillon R, 900, Rue St-Denis, Porte R10-464, Montréal, QC H2X 0A9, Canada; fredsaad@videotron.ca

**Keywords:** anti-neoplastic agents, cancer pain, prostatic neoplasms, quality of life, treatment outcome

## Abstract

**Simple Summary:**

Patients with prostate cancer often experience pain, fatigue and other negative symptoms that can lead to poorer quality of life. Enzalutamide is a prostate cancer therapy that is effective across the disease continuum from early-state cancer patients through to patients with metastatic castration-resistant disease. In this study, we evaluated how enzalutamide impacts patients’ quality of life. We found that patients with early disease maintained low pain levels and symptom-related burden when treated with enzalutamide or with a control treatment, and that patients with advanced disease who received enzalutamide experienced mitigated negative impacts compared to controls. Furthermore, it took longer for patients treated with enzalutamide to report experiencing a reduction in quality of life, and this was most pronounced for patients with advanced cancer. Enzalutamide can be tolerated by patients with early or advanced prostate cancer and delays both disease progression and the associated deterioration of quality of life.

**Abstract:**

This review examines the impact of treatment with enzalutamide on health-related quality of life (HRQoL) in prostate cancer patients across the disease continuum based on pivotal clinical trials. We assessed the effect of enzalutamide on pain, symptom burden and overall HRQoL from randomized controlled trials. Patient experience was evaluated in men with metastatic hormone-sensitive prostate cancer (mHSPC), non-metastatic castration-resistant prostate cancer (nmCRPC) and metastatic castration-resistant prostate cancer (mCRPC) (pre-chemotherapy and post-chemotherapy). Patients across the disease continuum reported a generally positive status at baseline, with relatively low levels of pain and impairment due to cancer-related symptoms and high HRQoL. For patients with earlier-state prostate cancer, pain and symptom-related burden were low at study entry and remained so, regardless of whether patients received enzalutamide or control treatment. Patients with more advanced disease reported mitigation in pain and symptom burden while receiving treatment with enzalutamide. Enzalutamide was observed to slow deterioration of overall HRQoL most for patients with nmCRPC or mCRPC (statistical significance for between-group difference in median time to deterioration: mHSPC (confirmed) *p* = 0.2998; nmCRPC (confirmed) *p* = 0.0044; mCRPC (unconfirmed) *p* < 0.0001). Across the prostate cancer continuum, enzalutamide is well-tolerated and delays the negative impact that disease progression has on quality of life.

## 1. Introduction

The androgen receptor inhibitor enzalutamide has demonstrated consistent benefits in men with prostate cancer at various stages of the disease, from hormone-sensitive prostate cancer (HSPC) to metastatic (m) and non-metastatic (nm) castration-resistant prostate cancer (CRPC) [1,2,3,4,5]. This has been established through several phase 3 clinical trials that have demonstrated the safety and efficacy of enzalutamide across the spectrum [1,2,3,4,5] and provided a unique opportunity to better understand the effect of treatment on the disease across a broad clinical spectrum.

In addition to safety and efficacy, measures of health-related quality of life (HRQoL) are increasingly important to patients and providers, and can be used to monitor treatment efficacy and disease progression [6,7]. HRQoL is influenced by disease symptoms, number and types of treatments used (and accompanying adverse events (AEs)) and evolving comorbidities [8]. In patients at earlier disease states who are relatively symptom-free, HRQoL is mostly influenced by AEs of local or adjuvant systemic treatments by anxiety related to recurrence or by other negative effects resulting from diagnosis without treatment when undergoing conservative management approaches (i.e., watchful waiting or active surveillance) [9]. As the disease metastasizes, patients may experience negative symptoms ranging from pain and discomfort to functional deficits caused by disease progression and the systemic treatments administered to control the disease [10].

This paper examines HRQoL data from four pivotal enzalutamide trials to present key insights into the patients’ HRQoL experience across disease states, ranging from mHSPC through to mCRPC in the post-chemotherapy setting. This review aims to highlight the impact of treatment with enzalutamide for prostate cancer on HRQoL measures that are meaningful to patients across the disease continuum.

## 2. Materials and Methods

This is a summary review of the patient-reported outcome (PRO) data from pivotal enzalutamide publications that used additional unpublished data to supplement published material (e.g., additional time points or subscale analyses). Detailed methods are provided in Appendix A. Complete study methods for the retrieved trials have been previously published [1,2,3,4]. Information concerning imputation and non-responses were addressed in previous publications, and non-responses were adjusted based on the scoring algorithms of the European Organisation for Research and Treatment of Cancer—Quality of Life questionnaire and Functional Assessment of Chronic Illness Therapy scoring guides. A brief description of the included studies is depicted in Table 1, with a focus on the PRO measures assessed in the trial.

### 2.1. HRQoL Instruments

The impact of enzalutamide on PRO measures across the disease spectrum, pain, symptom burden and overall HRQoL was assessed. The Functional Assessment of Cancer Therapy—Prostate (FACT-P), EuroQol 5-Dimension 5-Level questionnaire (EQ-5D-5L) and Brief Pain Inventory–Short Form (BPI-SF) were administered in the pivotal enzalutamide trials to evaluate the patient experience. For a detailed description of these measures and an overview of the frequency of administration of the FACT-P, EQ-5D-5L and BPI-SF across studies, refer to Appendix B, Table A1.

The FACT-P [8] is a self-reported measure that assesses HRQoL in patients with prostate cancer. It is composed of the Functional Assessment of Cancer Therapy—General, which measures various aspects of well-being applicable across oncology, and additionally includes the Prostate Cancer Subscale (PCS), which measures aspects specific to prostate cancer such as pain (i.e., PCS−Pain subscale), weight loss, urinary symptoms, bowel and bladder function and erectile dysfunction. Higher FACT-P scores indicate better outcomes for patients.

The EQ-5D-5L [16] is a standard self-reported measure that assesses health outcomes from a wide variety of interventions on a common scale for purposes of evaluation, allocation and monitoring. The questionnaire covers several domains: mobility, self-care, usual activities, pain/discomfort and anxiety/depression. Higher scores indicate better outcomes.

The BPI-SF [17] measures the extent to which pain interferes with mood, physical and social activity, work, relations with others and sleep, as well as current pain, worst pain, least pain and average pain levels. Higher scores on the BPI indicate higher levels of pain.

### 2.2. Statistical Analyses

Details of the statistical analysis used in the clinical trials can be found in corresponding publications [11,12,13,14,15], and a summary is provided in Appendix A. Clinically meaningful within-patient change thresholds for FACT-P, EQ-5D-5L and BPI-SF were based on previously established values [18,19,20], as reported in Table 2.

## 3. Results

### 3.1. Baseline Characteristics

Baseline characteristics of patients included in the different studies are provided in Table 3. Overall, sample sizes were large: PROSPER, PREVAIL and AFFIRM each included over 800 patients in the enzalutamide arm, while over 500 patients received enzalutamide in the ARCHES study. The median age of men recruited for the studies was approximately 70 years (range 41 years (AFFIRM) to 95 years (PROSPER)) across trials, broadly in line with population averages [21]. As expected, characteristics varied according to the disease state and respective inclusion criteria; for instance, more patients received local treatments in the CRPC state, while the proportion of patients with European Cooperative Oncology Group (ECOG) performance status scores of 1 was found to increase with disease state. It was anticipated that these differences may have implications for reported HRQoL.

Completion rates for PROs are found in Appendix B, Table A2. Median follow-up times for patients receiving enzalutamide and control treatment respectively, were 14.8 and 14.1 months (ARCHES), 18.5 and 15.1 months (PROSPER), 22.2 and 22.4 months (PREVAIL) and 14.4 and 14.4 months (AFFIRM).

### 3.2. Baseline HRQoL Scores

At study entry, patients across the disease continuum reported a generally positive outlook, with relatively low levels of pain, low levels of impairment due to cancer-related symptoms and high overall HRQoL (Table 4). Across the studies, patients reported highest scores (higher HRQoL/lower symptoms) in earlier states of the disease, and a gradual decline was observed coinciding with disease progression. Patients receiving chemotherapy for mCRPC indicated more problems than those at earlier states, reporting moderate pain and symptom burden. However, even patients with late-state prostate cancer entered the respective studies with few symptoms and good HRQoL.

### 3.3. Pain

**Figure 1:**
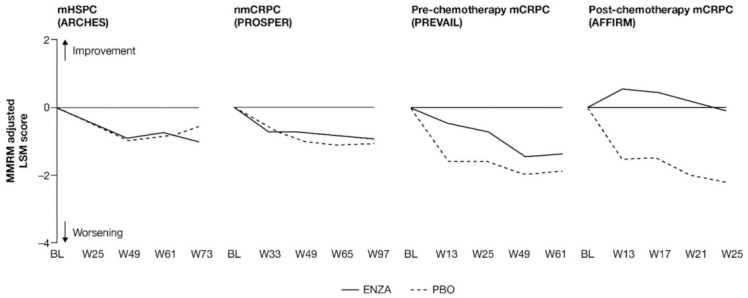
Adjusted change from baseline in PCS−Pain FACT-P subscale score. ENZA indicated by solid line, and control group indicated by dashed line. Abbreviations: BL: Baseline; ENZA: Enzalutamide; LSM: Least squares mean; mCRPC: Metastatic castration-resistant prostate cancer; mHSPC: Metastatic hormone-sensitive prostate cancer; MMRM: Mixed-model repeated measure; nmCRPC: Non-metastatic castration-resistant prostate cancer; PBO: Placebo; PCS: Prostate Cancer Subscale of the FACT-P; W: Week.

For patients with earlier-state prostate cancer, pain at study entry was low and remained so, while patients with more advanced disease reported a delayed progression of pain when receiving treatment with enzalutamide (Figure 1 and Appendix B, Table A3).

Patients in each trial/disease state reported mild-to-moderate levels of pain (mean PCS−Pain scores ranging from 13.2 (nmCRPC) to 9.7 (post-chemotherapy mCRPC) out of a total of 16) at baseline. For all subscales of the FACT-P, including the PCS−Pain subscale, note that higher scores indicate better HRQoL. For patients with earlier disease states (i.e., mHSPC and nmCRPC) receiving either enzalutamide or control treatment, pain scores remained close to baseline levels [11,12]. For poorer-prognosis patients with mCRPC, patients treated with enzalutamide maintained stable pain levels or, in the case of post-chemotherapy patients, experienced reduced pain levels, in contrast to control patients.

**Figure 2:**
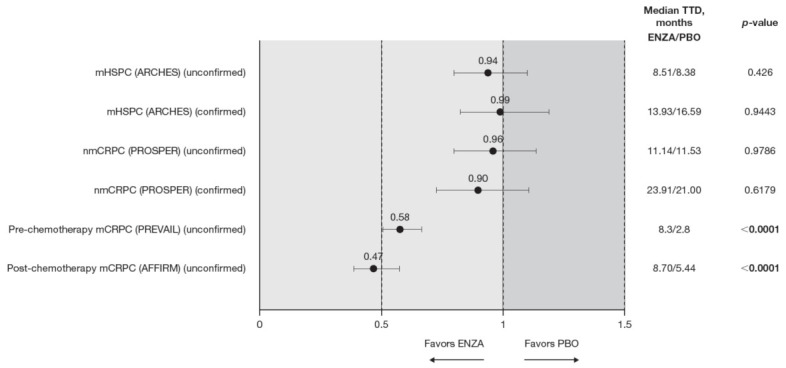
PCS−Pain FACT-P subscale score time to deterioration. To meet criteria for “confirmed” time to first deterioration, the initial (unconfirmed) report of deterioration had to be verified at the next consecutive study visit. Abbreviations: ENZA: Enzalutamide; mCRPC: Metastatic castration-resistant prostate cancer; mHSPC: Metastatic hormone-sensitive prostate cancer; nmCRPC: Non-metastatic castration-resistant prostate cancer; PBO: Placebo; PCS: Prostate Cancer Subscale of the FACT-P; TTD: Time to deterioration.

Analysis of change from baseline indicated statistically significant differences in pain scores between treatment groups, favoring enzalutamide in patients with CRPC (Figure 2). For patients with earlier-state disease, enzalutamide was not associated with a meaningful change in pain levels compared to control treatment. In contrast, for patients with mCRPC who had undergone chemotherapy, there was a pronounced advantage in favor of enzalutamide (higher symptom scores indicate lower levels of pain). While the score increased (improved) in the enzalutamide arm, a decrease (worsening) was observed for the control arm, with the difference between arms reaching clinically meaningful values at week 13. The least squares mean (LSM) change from baseline results observed with PCS−Pain scores was further supported by the results for BPI-SF worst pain (Appendix B, Table A3).

For pre-chemotherapy patients with mCRPC, there was no difference in PCS−Pain scores between treatment groups at the 61-week endpoint [13], although a significant difference was observed at weeks 5, 13, 25 and 37 (Figure 1). This difference at week 25 was corroborated by the BPI-SF (Appendix B, Table A3). In post-chemotherapy mCRPC (PCS−Pain data only), there was a difference in LSM change from baseline at 25 weeks, with patients receiving enzalutamide remaining stable, while control patients exhibited a clinically meaningful deterioration of more than 2 points. The difference between arms was statistically significant and clinically meaningful.

Time to pain progression as measured by the PCS−Pain score was significantly delayed in patients with mCRPC treated with enzalutamide (pre-chemotherapy and post-chemotherapy) compared to controls (Figure 2). Regardless of the definition of time to deterioration (confirmed or unconfirmed event), time to deterioration was generally comparable between groups for early-state patients (i.e., patients with mHSPC and nmCRPC) [11,12].

**Figure A1:**
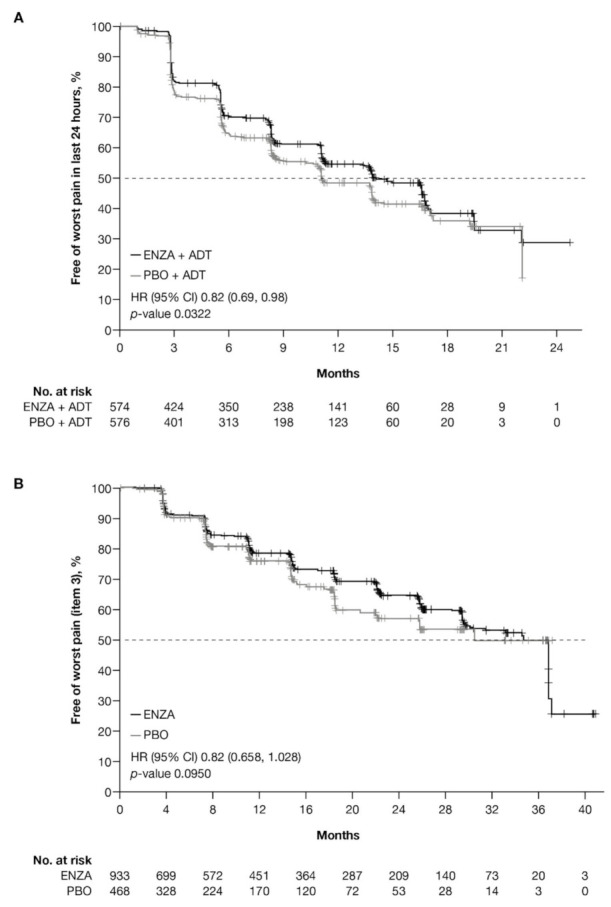
Kaplan–Meier curves for BPI-SF worst pain item. Free from BPI worst pain item (item 3) for (**A**) ARCHES trial patients with mHSPC and (**B**) PROSPER trial patients with nmCRPC. Abbreviations: BPI: Brief Pain Inventory; BPI-SF: Brief Pain Inventory–Short Form; CI: Confidence interval; ENZA: Enzalutamide; HR: Hazard ratio; mHSPC: Metastatic hormone-sensitive prostate cancer; nmCRPC: Non-metastatic castration-resistant prostate cancer; PBO: Placebo.

Data from the BPI-SF supported data from the PCS−Pain subscale: enzalutamide had a positive benefit on time to deterioration in the BPI-SF worst pain scores (Table 5). There was a trend in time to deterioration in BPI-SF worst pain score favoring enzalutamide across disease states, reaching statistical significance only in patients with mHSPC and pre-chemotherapy mCRPC. The benefit did not reach statistical significance (*p* = 0.085) for patients with nmCRPC. Data were not available for patients with post-chemotherapy mCRPC. Appendix B, Figure A1 presents Kaplan–Meier curves for BPI-SF worst pain.

### 3.4. Prostate Cancer Symptoms

Prostate cancer symptoms were evaluated using the FACT-P PCS. Prostate cancer symptoms appeared to be mitigated in patients with mCRPC receiving enzalutamide, while earlier-state patients receiving enzalutamide were more similar to control patients.

As with pain, patients across the studies tended to enter at baseline with relatively low symptom levels: the lowest mean symptom burden (34.7 out of 48) was reported by patients with nmCRPC, while the highest levels were found in patients with post-chemotherapy mCRPC. Results suggest that enzalutamide was most protective against increased symptom burden for patients with more advanced disease.

**Figure 3:**
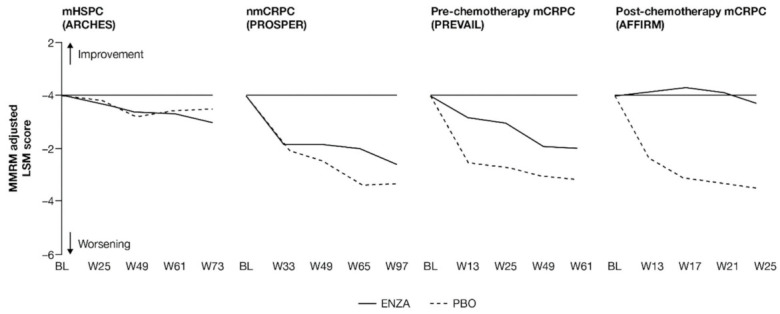
Adjusted change from baseline in FACT-P PCS score. ENZA indicated by solid line, and control group indicated by dashed line. Abbreviations: BL: Baseline; ENZA: Enzalutamide; FACT-P: Functional Assessment of Cancer Therapy—Prostate; LSM: Least squares mean; mCRPC: Metastatic castration-resistant prostate cancer; mHSPC: Metastatic hormone-sensitive prostate cancer; MMRM: Mixed-model repeated measure; nmCRPC: Non-metastatic castration-resistant prostate cancer; PBO: Placebo; PCS: Prostate Cancer Subscale of the FACT-P; W: Week.

FACT-P PCS scores show that enzalutamide mitigated prostate cancer symptoms in later states of prostate cancer (Figure 3 and Appendix B, Table A4). Adjusted LSM changes from baseline of symptom scores indicate that enzalutamide had a significant advantage over the control treatment in patients with mCRPC, with no significant differences for patients with mHSPC and nmCRPC.

For those patients with less-advanced disease (mHSPC or nmCRPC), no marked changes from baseline were observed between enzalutamide and controls, and prostate cancer symptom burden appeared relatively stable across the trials, regardless of treatment. The analysis demonstrated that no statistically significant changes from baseline in FACT-P PCS scores were observed at week 73 for patients with mHSPC or at week 97 for patients with nmCRPC in either of the treatment arms, indicating that both groups reported stable outcomes over time [11,12].

However, for later-state patients with mCRPC, particularly those who had undergone chemotherapy, enzalutamide limited the effects of progression on HRQoL and provided protection from symptoms and effects related to prostate cancer compared to the control treatment. In particular, for those with post-chemotherapy mCRPC, while symptoms improved in the enzalutamide arm, a worsening was observed for the control arm. For patients with mCRPC (pre-chemotherapy and post-chemotherapy), there was a statistically significant difference in PCS change from baseline between treatment groups at all time points. Furthermore, by the trial endpoints (weeks 61 (pre-chemotherapy PREVAIL) and 25 (post-chemotherapy AFFIRM)), the control group had experienced a clinically meaningful worsening, while the enzalutamide group had not [13]. Between-group differences were significant for mCRPC patients both pre-chemotherapy and post-chemotherapy.

**Figure 4:**
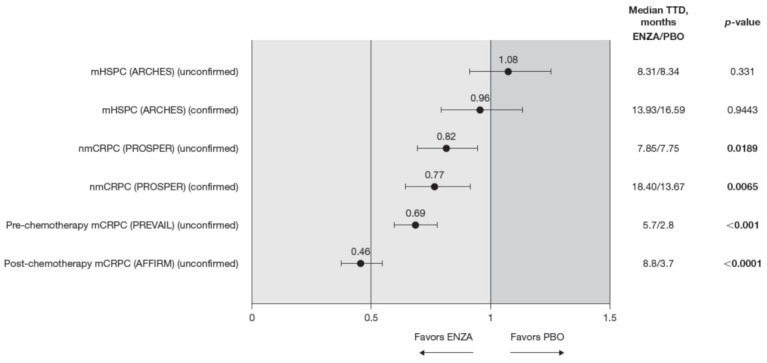
FACT-P PCS score TTD. To meet criteria for “confirmed” time to first deterioration, the initial (unconfirmed) report of deterioration had to be verified at the next consecutive study visit. Abbreviations: ENZA: Enzalutamide; FACT-P: Functional Assessment of Cancer Therapy—Prostate; mCRPC: Metastatic castration-resistant prostate cancer; mHSPC: Metastatic hormone-sensitive prostate cancer; nmCRPC: Non-metastatic castration-resistant prostate cancer; PBO: Placebo; PCS: Prostate Cancer Subscale of the FACT-P; TTD: Time to deterioration.

Compared to the control treatment, enzalutamide significantly delayed time to deterioration in FACT-P PCS scores for patients with nmCRPC and mCRPC, with no impact in hormone-sensitive patients (Figure 4). In patients with mHSPC, no significant differences were observed between enzalutamide and controls, regardless of whether the event was confirmed (hazard ratio (HR) 0.96) or not (HR 1.08) [11].

Enzalutamide significantly delayed time to deterioration of PCS scores vs. control treatment in patients with CRPC, regardless of the metastatic status of the disease, with an increased benefit (lower HR) as the disease progressed towards post-chemotherapy. Time to deterioration for the FACT-P PCS was prolonged by enzalutamide in patients with nmCRPC, as well as for those patients with mCRPC, both in the pre-chemotherapy and post-chemotherapy settings [12,13].

### 3.5. HRQoL

For overall well-being reported by patients, enzalutamide was observed to slow the deterioration of HRQoL, particularly for those with nmCRPC and mCRPC.

HRQoL scores followed similar trends to the previously outlined symptom scores: enzalutamide maintained high baseline HRQoL scores in a similar manner to those receiving androgen deprivation therapy (ADT) plus placebo in patients with earlier disease states (i.e., mHSPC and nmCRPC), while the protective effect of enzalutamide on HRQoL compared to control treatment was most noticeable in patients with mCRPC in the pre-chemotherapy and post-chemotherapy settings.

**Figure 5:**
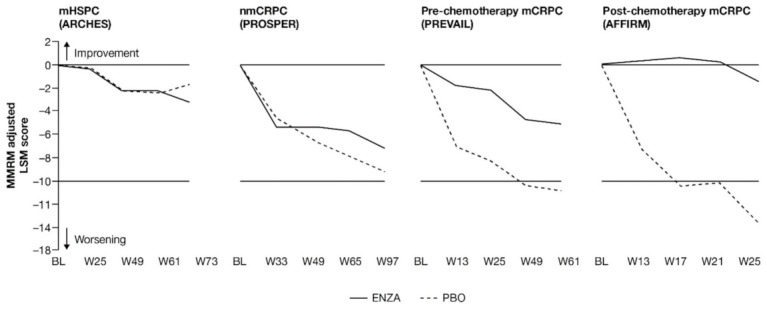
Adjusted change from BL in FACT-P total score. ENZA indicated by solid line, and control group indicated by dashed line. Abbreviations: BL: Baseline; ENZA: Enzalutamide; FACT-P: Functional Assessment of Cancer Therapy—Prostate; LSM: Least squares mean; mCRPC: Metastatic castration-resistant prostate cancer; mHSPC: Metastatic hormone-sensitive prostate cancer; MMRM: Mixed-model repeated measure; nmCRPC: Non-metastatic castration-resistant prostate cancer; PBO: Placebo; W: Week.

**Figure 6:**
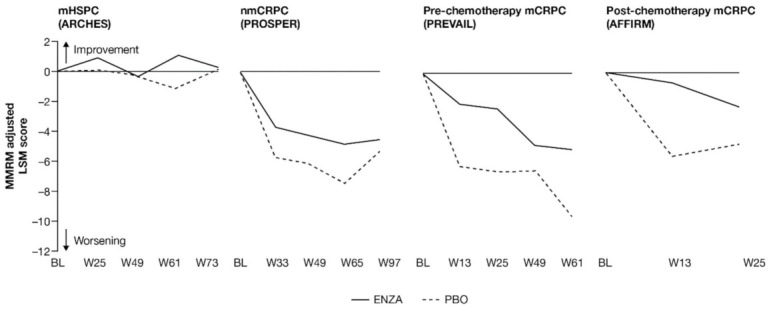
Adjusted and unadjusted change from BL in VAS score. ENZA indicated by solid line, and control group indicated by dashed line. For AFFIRM, EQ-5D-5L VAS was only collected at weeks 13 and 25. Abbreviations: BL: Baseline; ENZA: Enzalutamide; EQ-5D-5L: EuroQol 5-Dimension 5-Level questionnaire; LSM: Least squares mean; mCRPC: Metastatic castration-resistant prostate cancer; mHSPC: Metastatic hormone-sensitive prostate cancer; MMRM: Mixed-model repeated measure; nmCRPC: Non-metastatic castration-resistant prostate cancer; PBO: Placebo, VAS: Visual Analog Scale; W: Week.

Change from baseline in FACT-P total score and EQ-5D-5L Visual Analog Scale (VAS) score showed that enzalutamide maintained baseline HRQoL across the disease continuum, including in more advanced prostate cancer (Figure 5 and Figure 6). For less advanced patients, HRQoL, which was good at study entry, was maintained at similar levels regardless of treatment, while for patients with mCRPC, change from baseline favored enzalutamide over controls. For these later-state patients, those receiving enzalutamide maintained overall HRQoL at a level similar to baseline values, while those on placebo deteriorated sooner. This was observed both for FACT-P total scores and EQ-5D-5L VAS scores (Appendix B, Table A5).

Early-state patients reported relatively stable HRQoL, regardless of treatment. Although there was an early trend toward between-group differences in patients with nmCRPC, the change from baseline was comparable between groups at 97 weeks (Figure 6).

For pre-chemotherapy and post-chemotherapy patients with mCRPC [13,15], enzalutamide was not associated with a change from baseline, while a clinically meaningful deterioration was observed in the control arm. The difference between treatment arms was statistically significant [13,15]. For the post-chemotherapy patients with mCRPC, the difference between treatment arms was both statistically significant and clinically meaningful [15]. These results were corroborated by the VAS for the pre-chemotherapy group.

**Figure 7:**
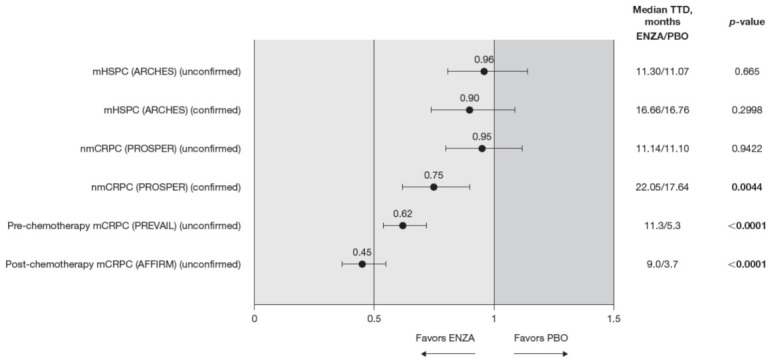
FACT-P total score TTD. To meet criteria for “confirmed” time to first deterioration, the initial (unconfirmed) report of deterioration had to be verified at the next consecutive study visit. Abbreviations: ENZA: Enzalutamide; FACT-P: Functional Assessment of Cancer Therapy—Prostate; mCRPC: Metastatic castration-resistant prostate cancer; mHSPC: Metastatic hormone-sensitive prostate cancer; nmCRPC: Non-metastatic castration-resistant prostate cancer; PBO: Placebo; TTD: Time to deterioration.

Across the disease continuum, there was a trend indicating that enzalutamide delayed time to deterioration of HRQoL as measured by the FACT-P compared to controls (Figure 7). This trend reached significance for patients with castration-resistant disease.

The HR for time to deterioration was consistently <1, i.e., favored enzalutamide over control treatment, regardless of whether the definition included the first confirmed or unconfirmed event. However, the delay in deterioration reached statistical significance only for unconfirmed time to deterioration in patients with CRPC. Patients with mHSPC on enzalutamide had a similar time to deterioration (determined by a 10-point decrease in the FACT-P) as control patients [11]. Time to deterioration for HRQoL, as measured by the FACT-P, was prolonged by enzalutamide in patients with nmCRPC (confirmed time to deterioration only) as well as for those patients with mCRPC, both in the pre-chemotherapy and post-chemotherapy settings [12,13].

**Figure 8:**
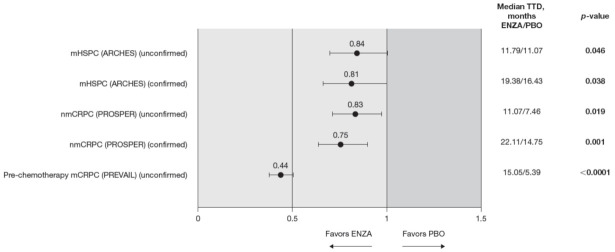
VAS score TTD. To meet criteria for “confirmed” time to first deterioration, the initial (unconfirmed) report of deterioration had to be verified at the next consecutive study visit. The EQ-5D-5L VAS was not collected in the AFFIRM pivotal trial. Abbreviations: ENZA: Enzalutamide; EQ-5D-5L: EuroQol 5-Dimension 5-Level questionnaire; mCRPC: Metastatic castration-resistant prostate cancer; mHSPC: Metastatic hormone-sensitive prostate cancer; nmCRPC: Non-metastatic castration-resistant prostate cancer; PBO: Placebo; TTD: Time to deterioration; VAS: Visual Analog Scale.

The trends observed in the VAS were generally in line with those observed for the FACT-P but are statistically significant, favoring enzalutamide (Figure 8).

## 4. Discussion

This study demonstrates that treatment with enzalutamide across the spectrum of prostate cancer, although associated with some AEs [1,2,3,4], does not worsen HRQoL compared to ADT alone [11,12,13,14,15]. Patients entering these trials with high HRQoL and low pain levels who received enzalutamide did not experience worsening of HRQoL or pain measures compared to control patients. Notably, enzalutamide was associated with significant HRQoL benefits in advanced disease, where patients may already experience a decrement in HRQoL.

Taken together, the results from the FACT-P, EQ-5D-5L and BPI-SF suggest that HRQoL, symptom burden and pain are likely to remain stable in patients with mHSPC and nmCRPC, but gradually deteriorate when patients progress to mCRPC. Treatment with enzalutamide may mitigate disease impacts for these later-state patients. This effect is most evident when examining time to deterioration. Conversely, earlier-state patients receiving enzalutamide experience a similar stability of symptom and function scores compared to control-treated patients. These results are broadly supported by the European Organisation for Research and Treatment of Cancer 25-Item Prostate Cancer questionnaire data in patients with mHSPC and nmCRPC, reported elsewhere [11,12].

Since patients with mHSPC and nmCRPC had relatively high HRQoL and low pain at study entry, it was difficult to identify significant improvements. In contrast, in patients with mCRPC for whom HRQoL has been shown to rapidly deteriorate in the natural disease course, enzalutamide significantly delays this deterioration over control treatment.

These results are in line with findings from a large UK-based population-based study of over 35,000 patients that observed that patients across the disease continuum tend to report similar HRQoL levels [22]. It has been shown that patients find that prostate cancer treatments generally do not impair HRQoL (although ADT is associated with increased fatigue and hormonal dysfunction), contrary to the assumptions of some medical professionals [22,23]. Moreover, improvement or maintenance of HRQoL is consistently observed for enzalutamide across the prostate cancer spectrum, as well as for other therapies such as abiraterone acetate (in mCSPC and mCRPC), darolutamide (in nmCRPC) and apalutamide (in nmCRPC and mCSPC), indicating that this class of androgen receptor-targeted therapies may be a good choice for qualifying patients with prostate cancer [24,25,26,27,28,29,30].

This study has several limitations. First, the selection criteria for patients in each of the pivotal trials were not the same across trials, making it more difficult to draw direct comparisons across studies. Additionally, the treatment history in PREVAIL and AFFIRM (the trials of patients with mCRPC) may not be aligned with future clinical practice. Furthermore, the time period for each pivotal trial differs as a consequence of the variable disease states (i.e., progression occurs more rapidly for patients with more advanced disease).

Second, patients with higher ECOG performance status scores were either excluded or, in the case of the AFFIRM trial, represented a minority of the overall patient cohort (nine and eight patients with an ECOG score ≥2 for enzalutamide and controls, respectively), which may have led to an overestimation of the HRQoL and an underestimation of pain (a limitation that is not unique to this set of clinical trials). Additionally, some trials (i.e., ARCHES) remain immature in their follow-up, where further follow-up time is needed to understand the relationship between treatment, progression and PROs.

Lastly, the endpoints included in the summary are not fully independent scores. Specifically, the FACT-P PCS includes the FACT-P PCS−Pain score, while the FACT-P total score includes the PCS. Therefore, it is possible that the advantages observed are not as diverse as implied by the terminology reflecting pain, prostate cancer symptoms and HRQoL.

This novel approach seeks to pragmatically respond to the needs of clinicians and decision-makers and is the first time, to our knowledge, that the patient experience of a particular prostate cancer therapy has been evaluated across the disease state continuum. Furthermore, the clinical trials in this review included a large international sample size deemed to be representative of patients globally, who, at baseline, reflected the characteristics of typical patients at each disease state. The studies also used the same comparator across trials (standard of care at the time of the study). Lastly, the questionnaires are validated in this population and have been widely used in other studies. Future studies may continue to assess the benefit of enzalutamide across prostate cancer disease states, for instance through post-marketing surveillance trials or registry studies.

## 5. Conclusions

In addition to efficacy and safety, treatment choices in medicine should consider the impact of the intervention on the quality of life of the patient. Enzalutamide is well-tolerated across the prostate cancer continuum and can reduce the negative impact that disease progression has on HRQoL. This is valuable information for patients, clinicians and other stakeholders involved in treatment management decisions globally.

## Figures and Tables

**Table 1:** Overview of key enzalutamide studies.

Study	Trial Description	Patient Population	Treatments *	Key Eligibility Criteria	Associated HRQoL Publications	HRQoL Tools Used	HRQoL Endpoints
ARCHES [1] NCT02677896	Multi-center, international, phase 3, double-blind, randomized, placebo-controlled clinical study enrolling patients from November 2013 to June 2017	mHSPC	Patients were randomized to receive 160 mg qd oral enzalutamide + ADT or matched placebo + ADT	Pathologically confirmed prostate adenocarcinoma, without neuroendocrine differentiation, signet-cell or small-cell features Hormone-sensitive metastatic disease, either de novo or after recurrence after prior local therapy, documented by a positive bone scan or metastatic lesions on CT or MRI	Stenzl, 2020 [11]	FACT-P EQ-5D-5L BPI-SF EORTC QLQ-PR25	W49 W73
PROSPER [2] NCT02003924	Multi-center, international, phase 3, double-blind, randomized, placebo-controlled clinical study enrolling patients from November 2013 to June 2017	nmCRPC	Patients were randomized 2:1 to receive 160 mg qd oral enzalutamide or matched placebo	Pathologically confirmed prostate adenocarcinoma without neuroendocrine differentiation, signet-cell features or small-cell features Rising PSA levels despite castration-associated testosterone levels (serum testosterone level ≤1.73 nm/L (0.50 ng/mL)) Received ADT with a gonadotropin-releasing hormone agonist or antagonist or underwent bilateral orchiectomy No previous or current evidence of metastatic disease as assessed by CT or MRI for soft-tissue disease and by whole-body radionuclide bone scanning	Tombal, 2019 [12]	FACT-P EQ-5D-5L BPI-SF EORTC QLQ-PR25	W49 W97
PREVAIL [3] NCT01212991	Multi-center, international, phase 3, double-blind, randomized, placebo-controlled clinical study enrolling patients from September 2010 to September 2012	Pre-chemotherapy mCRPC	Patients were randomized to receive 160 mg qd oral enzalutamide or matched placebo	Histologically or cytologically confirmed adenocarcinoma of the prostate with documented metastases PSA progression, radiographic progression or both in bone or soft tissue, despite receiving LHRH analog therapy or undergoing orchiectomy Serum testosterone level ≤1.73 nm/L (50 ng/dL) Continued use of ADT No previous cytotoxic chemotherapy, ketoconazole or abiraterone acetate	Loriot, 2015 [13]	FACT-P EQ-5D-5L BPI-SF (BL)	W25 W61
AFFIRM [4] NCT00974311	Multi-center, international, phase 3, double-blind, randomized, placebo-controlled clinical study enrolling patients from September 2009 to November 2010	Post-chemotherapy mCRPC	Patients were randomized 2:1 to receive 160 mg qd oral enzalutamide or matched placebo	Histologically or cytologically confirmed diagnosis of prostate cancer Castrate levels of testosterone (<50 ng/dL (1.7 nm/L)) Previous treatment with docetaxel Progressive disease defined according to PCWG2 criteria, including three increasing values for PSA or radiographically confirmed progression with or without a rise in PSA levels	Fizazi, 2014, Cella, 2015 [14,15]	FACT-P EQ-5D-5L BPI-SF (BL)	W13 W25

Abbreviations: ADT: Androgen deprivation therapy; BL: Baseline; BPI-SF: Brief Pain Inventory–Short Form; CT: Computed tomography; EORTC QLQ-PR25: European Organisation for Research and Treatment of Cancer 25-Item Prostate Cancer questionnaire; EQ-5D-5L: EuroQol 5-Dimension 5-Level questionnaire; FACT-P: Functional Assessment of Cancer Therapy—Prostate; LHRH: Luteinizing hormone-releasing hormone; mCRPC: Metastatic castration-resistant prostate cancer; mHSPC: Metastatic hormone-sensitive prostate cancer; MRI: Magnetic resonance imaging; nmCRPC: Non-metastatic castration-resistant prostate cancer; PCWG2: Prostate Cancer Working Group 2; PSA: Prostate-specific antigen; qd: Once a day; W: Week. * Across all studies, patients in both treatment groups received concomitant ADT; enzalutamide was administered until disease progression or unacceptable toxicity.

**Table 2:** Thresholds for meaningful deterioration.

HRQoL Instrument	Primary Threshold *
FACT-P total score	Decrease of at least 10 points [18,19]
FACT-P PCS	Decrease of at least 3 points [18,19]
FACT-P PCS−Pain	Decrease of at least 2 points [18,19]
EQ-5D-5L VAS	Decrease of at least 7 points [20]
BPI-SF item 3	Increase of at least 30% from BL

Abbreviations: BL: Baseline; BPI-SF: Brief Pain Inventory–Short Form; EQ-5D-5L: EuroQol 5-Dimension 5-Level questionnaire; FACT-P: Functional Assessment of Cancer Therapy—Prostate; NA: Not applicable; PCS: Prostate Cancer Subscale of the FACT-P; VAS: Visual Analog Scale. * The primary analysis for PROSPER assumed censoring not at random and used a different established threshold (i.e., an increase of at least 2 points); however, a sensitivity threshold of 30% from BL was also implemented and is used here to align with the primary thresholds conducted for ARCHES and PREVAIL. Insufficient BPI-SF data were collected for AFFIRM.

**Table 3:** Baseline patient characteristics.

Description	ARCHES [11]		PROSPER [12]		PREVAIL [13]		AFFIRM [14,15]	
	ENZA	PBO	ENZA	PBO	ENZA	PBO	ENZA	PBO
*n*	574	576	933	468	872	845	800	399
Age								
Median age, years (range)	70.0 (46–92)	70.0 (42–92)	74.0 (50–95)	73.0 (53–92)	72.0 (43–93)	71.0 (42–93)	69.0 (41–92)	69.0 (49–89)
Age cohorts, years, %								
<65	25.8	26.4			20.5	21.2		
65–75	44.6	44.3						
≥65					79.5	78.8		
<75			52	57			75	74
≥75	29.6	29.3	48	43			25	26
Region, %								
Europe	59.4	59.7	49	50	53.3	52.8	57.6	55.9
North America	15.0	13.4	15	13	25	24.62	32.9	33.1
Rest of world	25.6	26.9	36	37	21.7	22.6	9.5	11
Disease localization at screening, %								
Bone only	46.7	42.5	1.3	1.3	39.9	39.6	28	31
Soft tissue only	8.9	7.8	0.00	0.4	14.2	17.6	8	9
Bone and soft tissue	37.8	41.8	0.1	0.0	45.0	42.0	63	60
None			98.6	98.3	0.8	0.7		
Previous prostatectomy, %	12.5	15.5	25.08	29.70	25.9	26.6	34.6	30.6
Previous primary radiation therapy, %	16.4 *	16.7 *	32.58	33.76	39.0	39.1	37.5	41.9
Number of prior chemotherapy regimens								
1	17.9 ^†^	17.7 ^†^					72	74
≥2							28	26
ECOG, %								
0	78.0	76.9	80	82	67.0	69.2	37	39
1	21.8	23.1	20	18	33.0	30.8	54	53
≥2	0	0	0	0			9	8
Gleason score at diagnosis, %								
2–4			2.25	2.56	0.8	0.9		
5–7			52.63	49.15	48.6	46.9		
≤7							50	48
<8	29.8	32.5						
≥8	67.2	64.8	40.84	44.23	50.6	52.4	50	52
Unknown			4.29	4.06				
Missing			0	0			74	31

Abbreviations: ECOG: Eastern Cooperative Oncology Group; ENZA: Enzalutamide; PBO: Placebo. * Radiotherapy type (e.g., primary, salvage, palliative) not specified. ^†^ Prior docetaxel therapy.

**Table 4:** Mean HRQoL scores at BL.

Disease State	Pain				Prostate Cancer Symptoms		HRQoL			
	PCS−Pain		BPI-SF Item 3		FACT-P PCS		FACT-P Total		EQ-5D-5L VAS	
	ENZA	PBO	ENZA	PBO	ENZA	PBO	ENZA	PBO	ENZA	PBO
mHSPC [11]	12.36	12.08	1.80	1.77	33.4	32.5	113.9	112.7	74.4	74.2
nmCRPC [12]	13.16	13.56	1.24	1.01	34.67	35.47	119.5	120.8	76.2	77.5
Pre-chemotherapy mCRPC [13]	12.64	12.77	1.03	0.99	34.22	34.04	119.6	119.4	77.2	75.9
Post-chemotherapy mCRPC [14,15]	9.7	9.9	2.97	3.13	30.4	31.0	108.7	110.6	67.0	64.7

Abbreviations: BL: Baseline; BPI-SF: Brief Pain Inventory–Short Form; EQ-5D-5L: EuroQol 5-Dimension 5-Level questionnaire; FACT-P: Functional Assessment of Cancer Therapy—Prostate; mCRPC: Metastatic castration-resistant prostate cancer; mHSPC: Metastatic hormone-sensitive prostate cancer; nmCRPC: Non-metastatic castration-resistant prostate cancer; PCS: Prostate Cancer Subscale of the FACT-P; VAS: Visual Analog Scale. PCS−Pain FACT-P subscale ranges from 0 to 16, where higher scores indicate less pain. BPI-SF item 3 scale ranges from 0 to 10, where higher scores indicate more pain. FACT-P PCS scale ranges from 0 to 48, where higher scores indicate less interference of symptoms. FACT-P total score scale ranges from 0 to 156, where higher scores indicate better HRQoL. EQ-5D-5L VAS scale ranges from 0 to 100, where higher scores indicate better HRQoL.

**Table 5:** Time to deterioration for BPI-SF worst pain.

Disease State	Enzalutamide TTD, Months	Placebo TTD, Months	HR	*p*-Value
mHSPC [11]	14.09	11.10	0.82	0.032
nmCRPC [12]	34.69	30.52	0.82	0.085
Pre-chemotherapy mCRPC	5.65 *	5.55 *	0.62	<0.0001
Post-chemotherapy mCRPC	NR	NR	NR	

BPI-SF: Brief Pain Inventory–Short Form; HR: Hazard ratio; mCRPC: Metastatic castration-resistant prostate cancer; mHSPC: Metastatic hormone-sensitive prostate cancer; nmCRPC: Non-metastatic castration-resistant prostate cancer; NR: Not reported; TTD: Time to deterioration. * BPI-SF recorded at 13 and 25 weeks only.

## Data Availability

Researchers may request access to anonymized, participant-level data, trial-level data and protocols from Astellas-sponsored clinical trials at www.clinicalstudydatarequest.com. For the Astellas criteria on data sharing, see: https://clinicalstudydatarequest.com/Study-Sponsors/Study-Sponsors-Astellas.aspx.

## Appendix A

### Appendix A.1. Detailed Methods

Publications describing health-related quality of life (HRQoL) assessments were retrieved for the following trials that compared enzalutamide and androgen deprivation therapy (ADT) to placebo and ADT: ARCHES (NCT02677896), men with metastatic hormone-sensitive prostate cancer; PROSPER (NCT02003924), men with non-metastatic castration-resistant prostate cancer; PREVAIL (NCT01212991), men with pre-chemotherapy metastatic castration-resistant prostate cancer (mCRPC); AFFIRM (NCT00974311), men with post-chemotherapy mCRPC [1,2,3,4].

In addition to the previous pivotal trials, the STRIVE (NCT02456740), TERRAIN (NCT01288911) and ENZAMET (NCT02446405) trials have been conducted to support the use of enzalutamide in various prostate cancer populations. These studies were not included in this summary review because they used a different comparator (mostly bicalutamide on top of ADT alone) rather than ADT as the control treatment, which would make it difficult to draw comparisons across studies. Furthermore, the active comparators implemented in these trials were not considered standard of care, which might limit the relevance of any conclusions.

Gaps in the published literature were assessed, and in addition to published peer-reviewed manuscripts, unpublished data were retrieved from clinical study reports to report a more complete picture of the patient experience.

Descriptive statistics for observed scores for each treatment group and time point were presented for total and domain-level scores. Longitudinal change from baseline at specific time points was assessed using mixed-model repeated-measures analyses, controlling for baseline covariates and randomization factors. Time to deterioration analyses were also conducted. Two definitions for time to deterioration were used: unconfirmed time to deterioration (which was the initial observation of a deterioration) and confirmed time to deterioration (which required the initial deterioration to be verified at the next consecutive study visit). Time to first clinically meaningful deterioration (unconfirmed) and time to first confirmed clinically meaningful deterioration were used for symptom worsening/HRQoL deterioration, with their specific meaning depending on the domain analyzed.

### Appendix A.2. Additional Details for Measures Used in the Study

#### Appendix A.2.1. Functional Assessment of Cancer Therapy—Prostate (FACT-P)

The recall period is 7 days, and each item of the FACT-P is rated on a 5-level Likert-type scale, from “not at all” to “very much.” Of particular interest in this study are the FACT-P total score, the FACT-P Prostate Cancer Subscale (PCS) score and the FACT-P PCS–Pain score. These scales are used to capture how the symptomatology of advancing prostate cancer impacts the patient experience while undergoing treatment: FACT-P total score (39 items (27 cancer-specific and 12 prostate cancer-specific); scores range from 0 to 156, where higher scores indicate better HRQoL), PCS score (12 items; scores range from 0 to 48, where higher scores indicate better HRQoL) and PCS–Pain score (4 items (3 from the PCS module and 1 from the Functional Assessment of Cancer Therapy—General); scores range from 0 to 16, where higher scores indicate better HRQoL).

#### Appendix A.2.2. EuroQol 5-Dimension 5-Level Questionnaire (EQ-5D-5L)

The EQ-5D-5L was collected in all pivotal trials, except for AFFIRM. Each domain is rated on a 5-level Likert-type scale. A Visual Analog Scale (VAS) of the EQ-5D-5L was used in the current study to evaluate HRQoL: the VAS records patients’ self-rated health on a vertical scale with endpoints labeled “The best health you can imagine” and “The worst health you can imagine.” Scores range from 0 to 100.

#### Appendix A.2.3. Brief Pain Inventory–Short Form (BPI-SF)

The BPI-SF was not collected at all time points and across all studies. In PREVAIL, the BPI-SF was only collected at weeks 13 and 25, not at the week 61 endpoint, and in AFFIRM, the BPI-SF was only collected at week 13, not at the week 25 endpoint. Item 3 (worst pain in the last 24 h) responses range from 0 to 10, where higher scores indicate worse pain. It was selected to supplement results from the PCS−Pain score.

## Appendix B

**Table A1:** Frequency of assessment.

	W1 *	W5	W13	W17	W21	W25	W33	W37	W49	W61	W65	W73	W81	W97
ARCHES														
FACT-P	X		X			X		X	X	X		X		
EQ-5D-5L	X		X			X		X	X	X		X		
BPI-SF	X		X			X		X	X	X		X		
PROSPER														
FACT-P	X			X			X		X		X		X	X
EQ-5D-5L	X			X			X		X		X		X	X
BPI-SF	X			X			X		X		X		X	X
PREVAIL														
FACT-P	X	X	X			X		X	X	X				
EQ-5D-5L	X		X			X		X	X	X				
BPI-SF	X		X			X								
AFFIRM														
FACT-P	X		X	X	X	X ^†^								
EQ-5D-5L	X		X			X ^†^								
BPI-SF	X		X											

Abbreviations: BPI-SF: Brief Pain Inventory–Short Form; EQ-5D-5L: EuroQol 5-Dimension 5-Level questionnaire; FACT-P: Functional Assessment of Cancer Therapy—Prostate; W: Week. * W1 used as baseline. ^†^ Completed at week 25 and every subsequent 12 weeks.

**Table A2:** Completion rates for FACT-P across studies.

	FACT-P Completion Rate, *n/N* (%)							
	BL	W13	W17	W25	W49	W61	W73	W97
ARCHES								
ENZA	550/572 (96)	533/572 (93)		499/535 (93)	340/391 (89)	236/265 (89)	128/146 (88)	
PBO	553/574 (96)	529/574 (92)		487/530 (92)	298/332 (90)	191/213 (90)	101/115 (88)	
PROSPER								
ENZA	887/933 (95)		841/888 (95)		637/685 (93)			365/389 (94)
PBO	439/468 (94)		420/444 (95)		250/268 (93)			96/103 (93)
PREVAIL								
ENZA	865/872 (99)	821/835 (98)		756/777 (97)	619/643 (96)	528/554 (95)	429/457 (94)	182/192 (95)
PBO	834/845 (99)	643/653 (99)		372/387 (96)	177/185 (96)	118/129 (92)	80/87 (92)	27/28 (96)
AFFIRM								
ENZA	783/800 (98)	645/672 (96)		503/531 (95)	237/269 (88)	120/136 (88)	53/58 (91)	1/1 (100)
PBO	394/399 (99)	254/264 (96)		95/103 (92)	23/28 (82)	8/12 (67)	5/6 (83)	

Abbreviations: BL: Baseline; ENZA: Enzalutamide; FACT-P: Functional Assessment of Cancer Therapy—Prostate; *n*: Number of patients with evaluable response forms; *N*: Total number of patients available to be assessed at a given time point (evaluable questionnaires being those with sufficient data for the calculation of at least one subscale); PBO: Placebo; W: Week.

**Table A3:** Change from BL in pain scores.

	PCS−Pain			BPI-SF Worst Pain		
	LSM Change from Baseline ENZA	LSM Change from Baseline PBO	*p-*Value	LSM Change from Baseline ENZA	LSM Change from Baseline PBO	*p-*Value
mHSPC [11] 73 weeks	−1.01	−0.56	0.285	0.54	0.33	0.2854
nmCRPC [12] 97 weeks	−0.93	−1.06	0.668	0.52	0.73	0.353
Pre-chemotherapy mCRPC [13] 61 weeks	−1.37	−1.87	0.11	0.90 *	1.30 *	0.0022
Post-chemotherapy mCRPC [14,15] 25 weeks	−0.09	−2.21	<0.001	NR	NR	NR

Abbreviations: BL: Baseline; BPI-SF: Brief Pain Inventory–Short Form; ENZA: Enzalutamide; LSM: Least squares mean; mCRPC: Metastatic castration-resistant prostate cancer; mHSPC: Metastatic hormone-sensitive prostate cancer; nmCRPC: Non-metastatic castration-resistant prostate cancer; NR: Not reported; PBO: Placebo; PCS: Prostate Cancer Subscale of the FACT-P. PCS−Pain FACT-P subscale ranges from 0 to 16, where higher scores indicate less pain; BPI-SF worst pain scale ranges from 0 to 10, where higher scores indicate more pain. * BPI-SF recorded at 13 and 25 weeks only.

**Table A4:** Change from BL in prostate cancer symptom scores.

	FACT-P PCS		
	LSM Change from Baseline ENZA	LSM Change from Baseline PBO	*p*-Value
mHSPC [11] 73 weeks	−1.01	−0.50	0.449
nmCRPC [12] 97 weeks	−2.61	−3.32	0.189
Pre-chemotherapy mCRPC [13] 61 weeks	−1.99	−3.18	0.020
Post-chemotherapy mCRPC 25 weeks	−0.32	−3.53	<0.001

Abbreviations: BL: Baseline; ENZA: Enzalutamide; FACT-P: Functional Assessment of Cancer Therapy—Prostate; HRQoL: Health-related quality of life; LSM: Least squares mean; mCRPC: Metastatic castration-resistant prostate cancer; mHSPC: Metastatic hormone-sensitive prostate cancer; nmCRPC: Non-metastatic castration-resistant prostate cancer; PBO: Placebo; PCS: Prostate Cancer Subscale of the FACT-P. FACT-P PCS score ranges from 0 to 48, where higher scores indicate better HRQoL.

**Table A5:** Change from BL in HRQoL scores.

	FACT-P Total Score			EQ-5D-5L VAS		
	LSM Change from BL ENZA	LSM Change from BL PBO	*p*-Value	LSM Change from BL ENZA	LSM Change from BL PBO	*p*-Value
mHSPC [11] 73 weeks	−3.17	−1.71	0.429	0.283	0.186	0.9530
nmCRPC [12] 97 weeks	−7.17	−9.20	0.184	−4.57	−5.29	0.639
Pre-chemotherapy mCRPC [13] 61 weeks	−5.08	−10.87	<0.0001	−5.185	−9.764	0.0010
Post-chemotherapy mCRPC [15] 25 weeks	−1.5	−13.7	<0.001	−2.31	−4.80	0.487

Abbreviations: BL: Baseline; ENZA: Enzalutamide; EQ-5D-5L: EuroQol 5-Dimension 5-Level questionnaire; FACT-P: Functional Assessment of Cancer Therapy—Prostate; HRQoL: Health-related quality of life; LSM: Least squares mean; mCRPC: Metastatic castration-resistant prostate cancer; mHSPC: Metastatic hormone-sensitive prostate cancer; nmCRPC: Non-metastatic castration-resistant prostate cancer; PBO: Placebo; VAS: Visual Analog Scale. FACT-P total score scale ranges from 0 to 156, where higher scores indicate better HRQoL; EQ-5D-5L VAS scale ranges from 0 to 100, where higher scores indicate better HRQoL.
